# Use of nicotine replacement therapy to create a smoke-free home: study protocol for a pilot randomised controlled trial of a smoke-free home intervention in Scotland

**DOI:** 10.1136/bmjopen-2025-107161

**Published:** 2025-08-21

**Authors:** Rachel O’Donnell, Rebecca Howell, Tracy Henderson, Laura Sinclair, Karen Mather, Nicola McMeekin, Sean Semple

**Affiliations:** 1 Institute for Social Marketing and Health, Faculty of Health Sciences and Sport, University of Stirling, Stirling, Scotland, UK; 2 NHS Lanarkshire Tobacco Control Team, Lanarkshire, Scotland, UK; 3 Health Economics and Health Technology Assessment, School of Health and Wellbeing, University of Glasgow, Glasgow, Scotland, UK

**Keywords:** Tobacco Use, Smoking Reduction, Child protection, Family, Public health

## Abstract

**Abstract:**

**Introduction:**

The harmful health effects of children’s exposure to secondhand smoke (SHS) are well established. Most SHS exposure now occurs in the home, in low-income households. Previous research suggests that using nicotine replacement therapy (NRT) in the home can help with temporary smoking abstinence and could reduce smoking indoors. This pilot randomised controlled trial tests the feasibility of providing parents, carers and relatives with posted-to-home nicotine replacement therapy alongside fortnightly telephone support to reduce children’s exposure to SHS.

**Methods and analysis:**

100 participants are being recruited through existing National Health Service (NHS) Lanarkshire initiatives and social media. Parents/carers who are at least 18 years old, smoke in the home and care for one or more children aged 0–16 years are eligible to take part. Participants are randomised to either the intervention (Group A) or control (Group B) arm. Group A receives NRT posted to their home for 12 weeks free of charge, alongside fortnightly telephone calls and materials to support them in reducing children’s exposure to SHS. Group B is signposted to the Scottish Government’s ‘Take it Right Outside’ website which provides interactive advice on creating a smoke-free home. To quantify the child’s exposure to SHS, participants instal an air quality monitor to measure fine particulate matter (PM_2.5_) concentrations in their living room for 7 days at baseline and 12-week follow-up and/or collect and post saliva samples from their youngest child (age 5 or over) for cotinine analysis. Qualitative interviews explore intervention experience, NRT use and adherence and changes to home-smoking behaviours/smoking-related expenditure. Descriptive data analyses will be performed to address the feasibility of recruitment, randomisation, retention and adherence, data collection and intervention delivery. Analysis will also include pre/post changes (paired t-test) in both child’s salivary cotinine and PM_2.5_ levels to provide preliminary data on intervention effectiveness and difference between the intervention and control arms of the study. Health economics and resource use data will be collected and assessed for completeness, to test the process of data collection and estimate mean cost of both study arms.

**Ethics and dissemination:**

NHS ethical approval has been obtained by the West of Scotland Research Ethics Service (15 December 2023, ref 23/WS/0153; 13 December 2024, ref AM01). The findings will be disseminated to participants, funders, NHS Lanarkshire and other health services, and in peer-reviewed journals and academic conferences. Findings will inform new approaches that are timely and important, providing valuable evidence to help reduce children’s exposure to SHS in the home in Scotland and elsewhere.

**Trail registration number:**

ISRCTN79307718.

**STRENGTHS AND LIMITATIONS OF THIS STUDY**

- Our focus on intervention feasibility is important given many full trials face recruitment and retention challenges, potentially decreasing research value and the potential to influence policy and decision-making.  
- Parents, carers and relatives are able to participate in the study, which is a strength given relatives often play an important secondary caregiving role to families living in areas of high deprivation.
- Offering more than one means of measuring exposure to secondhand smoke will likely widen access and improve levels of recruitment and retention.
- Pregnant and breastfeeding women are excluded from participation, as the nicotine replacement therapy products offered are only licensed for this group of people who are making a quit attempt.
- Individuals who are not able to understand or speak English as their primary language are excluded from participating in the study, which is a limitation given the lack of adapted intervention programmes for migrants who wish to create a smoke-free home.

## Introduction

 The range of harmful effects associated with children’s exposure to secondhand smoke (SHS) is well established.^1 2^ Children exposed to SHS are at increased risk of many respiratory conditions including asthma, lower respiratory tract infection, croup and bronchiolitis.^3^ Creating a smoke-free home is key to reducing children’s SHS exposure and may also increase the likelihood of parents/carers quitting smoking.^4 5^ Growing up in a smoke-free home can also reduce adolescent smoking behaviours.^6^ As smoking is heavily concentrated in more disadvantaged groups, children from disadvantaged households face a higher risk of exposure to SHS.^7^

There is no guidance within Article 8 of the WHO Framework Convention on Tobacco Control on implementing measures to protect non-smoking adults and children from SHS in the home environment, while there is guidance for the 183 ratifying countries on protecting people from SHS in public places, workplaces and public transportation. This is stark given the home setting is where most children who breathe SHS receive their exposure.^8^ Systematic review findings conclude there is no consensus on how best to support families living in disadvantage to create a smoke-free home.^9 10^ Qualitative systematic review findings highlight that sole caring for young children in housing with limited/no access to private outdoor space constrains opportunities to smoke outside.^11^ In Scotland, this is reflected in recent Scottish Health Survey (SHeS) data, which showed that only 3% of children were exposed to SHS in their own home on a regular basis in 2022. However, 9% of children exposed to SHS at home lived in the most deprived areas of Scotland, compared with less than 1% of children living in Scotland’s most affluent areas.^12^ Inequalities in SHS exposure have been documented in several other countries including the USA,^13^ Australia,^14^ Germany,^15^ Spain,^16^ Denmark^17^ and Japan.^18^ In 2023, 4% of children living in Scotland were exposed to SHS in their own home on a regular basis;^19^ however, this still equates to over 30 000 children experiencing inequalities, who are exposed to a preventable carcinogen in the home. Collectively, these findings reinforce the need for innovative approaches to intervention development to better support disadvantaged parents to create a smoke-free family home.^20 21^

Previous studies conducted in Scotland suggest it is feasible to engage with people who smoke living in low-income areas to support them to create a smoke-free home, but behavioural change is often difficult due to a lack of opportunities to take smoking right outside and others (coresidents or visitors) smoking in the home.^20 22^ One possible means to overcome this is through use of nicotine replacement therapy (NRT) for temporary abstinence from smoking to avoid exposing others to SHS in the home. In Scotland, free access to NRT is only available for smoking cessation, although existing National Institute for Health and Care Excellence guidelines recommend NRT use for temporary abstinence inside the home.^23 24^ A recent randomised controlled trial (RCT), conducted with parents living in deprived communities in Nottingham, England, tested the effectiveness of a 12-week smoke-free homes intervention consisting of behavioural support, NRT for temporary abstinence (provided by a specialist smoke-free home adviser through home visits) and ongoing feedback on SHS exposure levels using air quality monitoring. Significant decreases were found in SHS concentrations and cigarettes smoked in the home for the intervention group compared with a ‘usual care’ group,^25^ and cost-effectiveness was demonstrated,^26^ but the specific effects of NRT for temporary abstinence remain unclear based on the findings from this multicomponent intervention. To explore the sole use of NRT to reduce children’s SHS exposure in the home, two pilot studies have been conducted with mothers, fathers and caregivers living in low-income areas in Edinburgh, Scotland.^27 28^

The first pilot study^28^ consisted of two phases, conducted in collaboration with Early Years Centres (EYCs), Family Nurse Partnership (FNP) centres and community pharmacies in Edinburgh. In Phase 1, qualitative interviews with 17 parents explored smoke-free home interventions that would better accommodate their circumstances. Parents supported the idea of NRT use for temporary abstinence to create a smoke-free home, viewing this as a safer option than using e-cigarettes indoors. In Phase 2, 32 parents were recruited from EYCs and discussed NRT product choice during a home visit from a smoking adviser. 20 collected their free NRT from the pharmacy, using it for up to 12 weeks in the home. During qualitative interviews, parents reported a range of outcomes including creating a smoke-free home, and/or reduced cigarette consumption by 50% or more in the home, and in a few cases quitting smoking. They also reported financial benefits and spending more time with their children. They often exceeded their own expectations of their ability to make changes to their smoking behaviour. NRT products were sometimes informally ‘shared’ with partners, fitting with recent calls for consideration of an ‘all household’ approach to smoke-free home interventions.^20 29^ While interviews with pharmacy, EYC and FNP staff (N=13) provided support for this novel approach, the multistep process used to access NRT was cumbersome and some participants did not engage due to this perceived barrier.

In Pilot study 2,^27^ 25 parents and caregivers (eg, grandparents) were recruited during the COVID-19 pandemic. Participants accessed their 12-week supply of NRT directly through community pharmacies, eliminating the need for an initial home visit from a smoking adviser. Indoor air quality was assessed at baseline and after 12 weeks as an objective measure of SHS reduction in the home. During qualitative interviews, a few participants reported creating a smoke-free home and one quit smoking. Some reduced cigarette consumption by 50% or more in the home while others reported no changes. Participants suggested telephone support to use NRT could assist behavioural change, and that NRT access could be streamlined by posting products to the home, thus addressing barriers to intervention uptake including reduced mobility, lone parenting and stigma associated with in-person discussions about smoking. These findings highlight the need to modify the intervention to provide ongoing support for NRT use, easier access to NRT and assess NRT adherence more robustly.

### Objectives

The aim of this study is to conduct a pilot RCT of use of free posted-to-home NRT alongside behavioural support by telephone to reduce children’s exposure to SHS in the home, to inform the development of a future definitive trial.

#### Primary objective

1. To analyse and report data on recruitment, randomisation, retention and adherence, data collection and intervention delivery.

#### Secondary objectives

1. To explore the experiences of participants and staff involved in intervention delivery regarding the pilot intervention and future trial design, including how our conceptual model, intervention content and data collection methods could be refined.
2. To pilot primary and secondary outcome measures (see below) and collection of resource use and economic data prior to a future definitive trial.

### Research questions 

To meet study objectives, we are using mixed methods to address these research questions: 

1. Is the intervention feasible in terms of participation and retention rates, NRT adherence and the practicalities of intervention delivery within an established National Health Service (NHS) service? (Objective 1) 
2. What are participant (and study team members’) experiences of the intervention and views about its potential impacts on home smoking in the home and smoking consumption? (Objective 2) 
3. What are barriers to, and facilitators of, implementation? (Objective 2) 
4. Is it feasible and acceptable to measure pilot trial primary and secondary outcomes of interest? (Objective 3) 
5. Is it feasible to collect the resource use cost-effectiveness data required to conduct a full health economic analysis of this intervention in a future trial? (Objective 3) 
6. Is it feasible for participants to collect saliva samples from their youngest child (age 5 or over) for cotinine analysis in preparation for a future trial? (Objective 3)

## Methods and analysis

### Recruitment and study design

Participants living in Lanarkshire, Scotland are being recruited by NHS intervention delivery staff via third sector and voluntary organisations. NHS Lanarkshire is the third largest health board in Scotland, responsible for the healthcare of more than 600 000 people living within the council areas of North Lanarkshire and South Lanarkshire. Lanarkshire has higher than average levels of deprivation, more children living in poverty and life expectancy is below national levels.^30^

**Figure 1:**
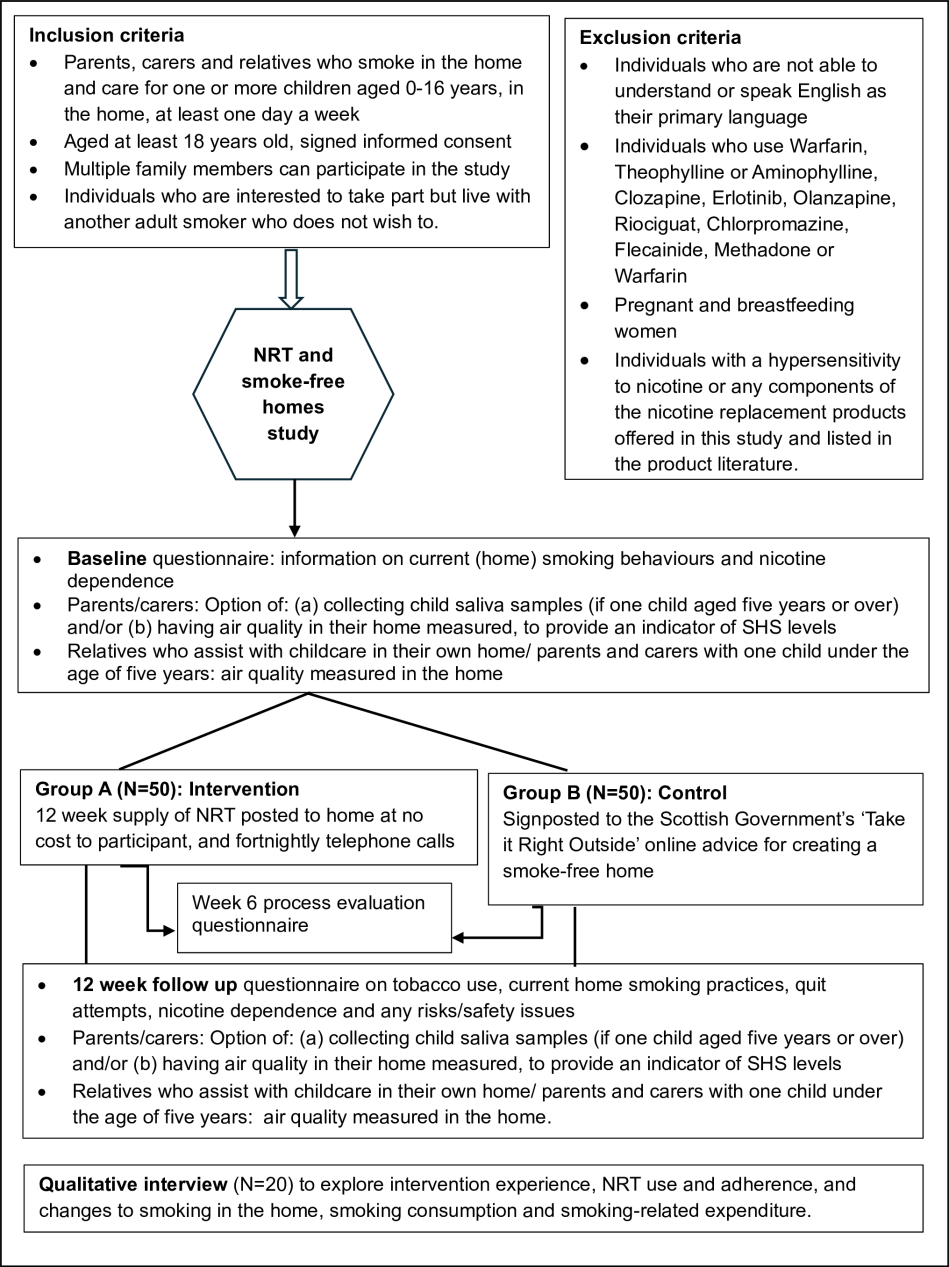
Study design. NRT, nicotine replacement therapy; SHS, secondhand smoke.

A range of techniques are being used to maximise the identification and recruitment of participants, including distribution of information sheets and flyers during prearranged visits to community venues, service centres and promotion/presentation of the study at staff team meetings. We are using social media advertisements to widen reach, having used this approach in three previous smoke-free home studies.^27 31 32^ We are monitoring instances where individuals ‘tag’ friends to alert them to the study, as per our previous studies, to ensure that use of Facebook does not lead to skewed sampling via self-selection of recruitment of friends. Participant recruitment started in January 2024 and is expected to end in September 2025. Figure 1 summarises the design of the pilot RCT and each of the trial aspects is described in detail.

### Eligibility criteria

Parents, carers and relatives who are at least 18 years old, smoke in the home and care for one or more children in the home, aged 0–16 years, at least 1 day per week, are eligible to participate. Multiple family members can participate in the study. Individuals who are interested in taking part but live with another adult smoker who does not wish to, are able to participate. SHS exposure is additive and linear—so we will be able to identify reductions in the number of cigarettes smoked through reductions in fine particulate matter (PM_2.5_) and/or salivary cotinine, even if another adult continues to smoke in the home. While obtaining a completely smoke-free home would not be viable in such cases, it would still be possible to reduce children’s exposure to SHS, which aligns with the aim of the study. Individuals who are not able to understand or speak English as their primary language are excluded given we do not have a budget to recruit, train and pay bilingual staff.

Individuals who use warfarin, theophylline or aminophylline, clozapine, erlotinib, olanzapine, riociguat, chlorpromazine, flecainide or methadone are excluded as NRT use would require general practitioner monitoring. Pregnant and breastfeeding women are excluded from participation as the NRT products offered are only licensed for this group of people who are making a quit attempt. Participants who have a hypersensitivity to nicotine or any components of the nicotine replacement products offered in this study and listed in the product literature are excluded from taking part—we establish this by asking specific questions regarding any previous use of NRT and whether participants have any allergies.

Reasons for participant ineligibility are recorded, and an informed consent process is followed during recruitment. Eligible individuals read the information sheet, or have it read to them, detailing the purpose of the trial and study procedures, potential benefits and risks of participation, data handling procedures, participant rights and relevant contact details. In the event that an individual loses capacity during participation in this research, we will retain all data up to the point of removing the participant from the study. This is also made clear in the participant information sheet.

Eligible individuals are also given a study privacy notice to read detailing how the information they provide is being collected, used and stored. Following informed consent procedures, after 48 hours of receiving these materials, a member of the NHS intervention delivery team telephones eligible individuals to see if they are willing to participate. Those who agree and are asked to complete the study consent form (see online supplemental file 1) and a short baseline questionnaire during this call to provide information on current (home) smoking behaviours.

Parents have the option of: (1) collecting child saliva samples and/or (2) having air quality in their home measured, to provide an indicator of SHS levels. Relatives who assist with childcare in their own home are not asked to obtain child saliva samples, as consent would be required from parents, but they are asked to monitor air quality in the home.

In cases where parents care for one child only, and they are under the age of 5, we measure air quality in the home, but we do not obtain saliva samples. Obtaining these samples from children below the age of 5 is more challenging and introduces potential hazards associated with them swallowing saliva sample equipment. If participating parents also have a child aged 5 years or older living with them in the home, we offer the option of saliva sampling involving this child and/or air quality monitoring.

Following methods used in the annual health survey in England for collection of child saliva samples, if parents choose this option, they are required to provide written consent for the collection of saliva samples from their youngest child aged 5 or over. A copy of the completed consent form is posted to all participants. We provide each participant with an age-appropriate information sheet by post for them to share and discuss with their child in advance of a prearranged home visit from a member of the NHS intervention delivery team. During this visit, the child is required to assent to the sample collection for it to proceed. The process for gaining assent from the child also follows those used in the annual health survey in England. Parents are required to give fully informed consent. Assent from children indicates that they have been given an age-appropriate explanation that they can understand (even if not as comprehensive as for an adult), and that the child is happy for the procedure to go ahead.^33^ Children are not required to sign anything, but the NHS Lanarkshire intervention delivery team member signs an ID form to confirm the child’s assent has been obtained during the home visit. With permission, (and in the case of parents, if they choose to have air quality measured in their home), the team member also instals an air quality monitor during this visit, to measure PM_2.5_ levels in the home for 1 week.

A suitable date/time is agreed during this home visit for the same team member to telephone each participant after 1 week, to initiate intervention delivery. Participants are randomised (1:1) to either the intervention (Group A) or control (Group B) arm prior to this telephone call. If more than one individual from the same family participates in the study, each family member is allocated to the same study arm.

### Sample size

We have drawn on published work to guide our sample size, which outlines a median sample size per arm of 36 for pilot RCTs with similar primary outcome measures.^34^ We have also considered the relatively homogenous nature of our sample in terms of deprivation and ethnicity, our intention to conduct qualitative interviews with low and high adherers, resources and costs. On this basis, we aim to recruit 100 participants, to have approximately 35 in each group (allowing for a 30% drop-out rate). 

### Intervention

The team member discusses NRT product choice with Group A participants using an information sheet which outlines the different types of NRT products available to them, the pros and cons of each and how to use them effectively. This information sheet builds on feedback from our pilot study with two participants on ways to enhance the intervention.^27^ The team member and Group A participants also discuss knowledge/understanding of SHS risks, how to change home smoking behaviours with support from other people who smoke in the household and the development of a personalised plan to identify and address barriers to creating a smoke-free home/ways. These discussions are aided by use of theory-and-evidence-based AFRESH materials,^35^ developed using intervention mapping, a rigorous framework which is widely used for developing complex behaviour change interventions. The AFRESH materials were designed to support people who smoke to plan to create a smoke-free home and engage support from others who smoke in the home.

The team member then arranges a 1-week supply of their chosen NRT product to be posted to their home alongside their completed personalised plan, hard copy of AFRESH leaflets and NRT product information sheet. Each participant receives a follow-up call at the end of week 1 of NRT use to establish product suitability; an alternative NRT product can be supplied if required. Once product suitability is established, 2–3 week supplies of NRT product are posted following an agreed protocol for provision. Fortnightly telephone support includes review (and revision) of personalised plans to create a smoke-free home and adherence to NRT. NHS Lanarkshire’s postal service for NRT products is already established within the Specialist Stop Smoking Service. A recent trial has suggested that posting NRT in conjunction with behavioural support is a practical, safe and inexpensive strategy in a cessation context.^36^

### Control arm

The team member signposts Group B participants to the Scottish Government’s ‘Take it Right Outside’ advice (https://www.nhsinform.scot/self-help-guides/second-hand-smoke-your-smoke-free-tips) by email. This NHS Inform website provides tailored interactive advice on creating a smoke-free home. On completion of the study at week 12, all participants in the control arm are offered AFRESH materials and the NRT product information sheet is discussed by telephone, alongside the offer of 12-week supply of free NRT products posted-to-home fortnightly.

With participant permission, Group A and Group B participant contact details are shared with a University of Stirling research team member to facilitate completion of the process evaluation questionnaire (Week 6) and recruitment to qualitative telephone interviews (Week 13). If a participant (in either arm) decides to quit smoking during the study, they are given the telephone number for NHS Lanarkshire’s specialist stop smoking service, letting them know that there is additional support available for their quit attempt and that the service could offer them alternative NRT products (ie, patches) if required. Indoor air quality and/or child salivary cotinine are still measured at follow-up, alongside administration of the 6-week process evaluation questionnaire (if applicable). If a participant in the intervention arm decides to quit smoking during the study, they are still contacted fortnightly to ascertain whether their quit attempt is still in progress or whether they would like to obtain further NRT supplies in order to create/maintain a smoke-free home. These calls continue to emphasise the benefits of reducing children’s exposure to SHS in the home, and the importance of ensuring that visitors to the home are not smoking indoors.

All participants (including those who decide to quit smoking during the study) are recontacted to obtain follow-up information during week 12, including on tobacco use, current home smoking practices, heaviness of smoking (Heaviness of Smoking Index), quit attempts, nicotine dependence and any risks/safety issues.

### Objective SHS measures

Parents and carers have the option of collecting child saliva samples and/or having air quality measured in their home, unless they care for one child in the home under the age of 5, in which case we will measure air quality in their home. We are also measuring air quality in the home of non-parental carers who agree to take part, but they do not have the option of obtaining saliva samples from the child, because parental consent is required for this.

We are assessing the feasibility of participants collecting and posting saliva samples from their youngest child (aged 5 or over) for cotinine analysis. Biological markers of SHS exposure are viewed as the ‘gold standard’,^37 38^ and salivary cotinine is widely accepted as the best biological indicator of SHS exposure. It is commonly collected in studies of children’s exposure to SHS in the home. It provides information on recent (hours–days) exposure to SHS, so it is well suited to our study given our baseline to follow-up period spans 12 weeks, and it can also quickly reflect change in household smoking behaviours. Other research^39^ has suggested that posted cotinine samples may be a reliable option for verifying adult tobacco use, and COVID-19 has made careful self-collection of biological samples using detailed instruction a much more common task among the public. If self-collected, posted saliva cotinine samples can be demonstrated to be a reliable way to gather data on SHS exposure, we will use this lower-cost method of collection in our future definitive trial, using a reduction in children’s salivary cotinine levels (comparing samples taken at baseline and 12-week follow-up) as our primary outcome measure. This finding would have also benefits for other work, including the SHeS which currently involves in-person visits for saliva collection.

Changes in indoor PM_2.5_ levels between baseline and follow-up (collected by air quality monitors) could provide an alternative method of determining changes in smoking behaviours in a larger trial, so we are also assessing the feasibility of participants measuring air quality and posting air quality monitors. We obtain consent from participants to measure indoor air quality for a 1-week duration, using a PurpleAir Flex air-quality monitoring device, at baseline and follow-up. The team member oversees participant installation of the air quality monitor during the initial home visit, using simple instructions for self-installation we have developed in our previous studies. Participants are asked to switch it on in the main living room of their home for a 1-week duration, to gather data on in-home PM_2.5_ concentrations at baseline (measuring PM_2.5_ is an internationally recognised, widely used method of quantifying SHS in the air).^38^ At the end of week 1, they receive a prearranged call from the researcher reminding them to return the monitor via a second prepaid postage box or a researcher visit if more convenient. During the home visit, parents who choose to also collect a sample of saliva for analysis of cotinine levels from their youngest child (aged 5 or over) use printed instructions (without input from the researcher). The team member collects a second sample from the same child immediately afterwards, essentially providing training to the participant on how to collect the sample for the follow-up sample in 12 weeks’ time. Each participant is asked to post the saliva sample they obtain back to the study team to test the feasibility of this part of the process. The study team member takes the saliva sample they obtain with them at the end of the home visit.

If initial evidence (after approximately 10 weeks of baseline data collection) suggests that these baseline, self-collected samples are returned through the post (>90%) and have sufficient saliva volume (>90%), this will provide us with the option of participants collecting and posting the second sample of saliva from the same child at follow-up. If these criteria are not met, we will gather data through in-person home visits and team member sample collection.

Each participant obtaining air quality measures in the home also receives the air quality monitor again at follow-up, and a second 1-week duration of measurement will be taken. All sampling/monitoring equipment is posted to the home at no expense to participants. Sampling sheets are used to record sample/monitor ID number (also on the saliva sample tube), the participant ID number and to confirm the child’s assent was obtained for providing saliva samples. We compare PM_2.5_ concentrations at baseline and Week 13. Participants can opt in to receive a lay summary of findings, including average baseline and follow-up salivary cotinine levels and PM_2.5_ levels for each group, at the end of the study. This lay summary will be co-designed with input from our public involvement groups. All participants receive two £25 supermarket vouchers of their choice for taking part in the study—the first after completing the 6-week process evaluation questionnaire by telephone, and the second at the end of the 12-week study. 

20 Group A and 5 Group B participants are taking part in qualitative telephone interviews conducted from week 13 to explore intervention experience, NRT use and adherence, and changes to smoking in the home, smoking consumption and smoking-related expenditure.

### Pilot outcome measures and progression criteria

The following data will be analysed to address the primary (feasibility) objectives of the pilot trial: 

- Recruitment, randomisation and retention: Number of participants screened, eligible and successfully recruited into the study, and the characteristics of non-consenting and ineligible participants; number of participants enrolled in the allocated recruitment time; retention of participants in their trial arms following randomisation, the proportion engaging in 12-week follow ups and number/reasons for dropping out. 
- Intervention delivery: The proportion of participants: (1) receiving NRT and demonstrating their adherence to NRT at fortnightly follow-ups; (2) using their personalised plan to identify and address barriers to creating a smoke-free home; and (3) engaging in fortnightly telephone support calls. 
- Data collection methods: Proportion of completed baseline and follow-up assessments in each arm; number of participants successfully providing child saliva samples and measuring air quality at baseline and follow-up. 

Our final range of secondary outcomes includes number of cigarettes smoked per day (in general and in the home), home smoking practices, self-reported quit attempts and nicotine dependence. 

We have developed progression criteria with input from our Study Advisory Group members, in line with current guidance.^40^ We are using the red/amber/green traffic light system^41^ to guide the decision on whether to progress to a definitive future trial using the criteria presented in table 1.

**Table 1:** Progression criteria.

% of sample size recruited	100% (35 in each arm)	70% or more (25 in each arm)	Less than 70%
% of follow ups completed (SHS measures)	70%	60–69%	Less than 60%
% of participants who try using NRT and engage with at least one fortnightly telephone call	70%	60–69%	Less than 60%
% reporting risks/safety concerns	None	See note 1	See note 1

The nature of reported risks and/or safety concerns could vary and include low, medium and high risks. On this basis, rather than specifying numbers for amber and red criteria, any reported risks/safety concerns will be discussed by the team and in conjunction with the Study Advisory Board to identify the extent to which they pose a concern in the context of decision making regarding a future definitive trial.

NRT, nicotine replacement therapy; SHS, secondhand smoke.

The decision on whether to progress to a definitive future trial will be guided by a combination of the progression criteria, important information learnt from the process evaluation questionnaire and the qualitative interviews and emerging evidence (eg, regarding the use of NRT products for temporary abstinence).

### Process evaluation, resource data and costs

All participants complete a short telephone questionnaire during week 6 of the intervention with acceptability questions on pilot-trial processes, any risks/safety issues and engagement with the intervention. Key issues are being further explored in qualitative interviews. The intervention delivery team records views on feasibility, experience of delivering the intervention and suggestions for improving study processes in a reflective diary. We are collecting resource data and assessing the feasibility of collecting data required for a cost-effectiveness analysis in a future trial, following current health economic analysis guidelines for pilot intervention studies.^40^ We have conducted a literature review to identify approaches and methods used for economic evaluations in similar studies and have identified potential costs and outcomes using a cost-consequences analysis framework. We have developed a conceptual model to visually represent the research problem and have used this to identify relevant resources used in the provision of the intervention including NRT type, dose and frequency; the staff grade and time spent providing telephone support. We have developed a resource use form to collect this data. 

### Data analysis plan

Descriptive data analyses will be performed to address the feasibility objectives. Child saliva samples will be processed for salivary cotinine by a specialist, accredited laboratory. The research team will analyse data from the air quality monitors. We will report the percentage of participants that return a valid (sufficient saliva) sample through self-collection, and the level of agreement between the self-collected and researcher-collected salivary cotinine values at baseline to determine the suitability of self-collection of these samples. Analysis will also include pre/post changes (paired t-test) in both child’s salivary cotinine and PM_2.5_ levels to provide preliminary data on intervention effectiveness and difference between the intervention and control arms of the study. Data on variability in these objective measures will also help calculate suitable sample size for the future trial. The qualitative data from the 6-week process evaluation questionnaire will be analysed using content analysis. All qualitative interviews will be recorded with participant permission, transcribed, anonymised and analysed using the framework approach. Transcripts and recordings will be stored securely and destroyed in line with University of Stirling procedures, taking account of General Data Protection Regulation. The health economics and resource use data will be assessed for completeness, to test the process of data collection and the mean cost of both study arms estimated. We will assess suitable outcomes and identify key drivers of uncertainty for any future economic evaluation in a large-scale trial. We will consider the suitability of data to inform a cost-consequence analysis (particularly suitable for public health interventions with multiple possible benefits) and conduct this analysis, to provide information on where further focus might be beneficial in a future definitive trial. 

### Ethics and dissemination

The study obtained ethical approval from the West of Scotland Research Ethics Service 3 (15 December 2023, ref 23/WS/0153; 13 December 2024, ref AM01). The study protocol is registered with the ISRCTN registry (ISRCTN79307718). The protocol and this manuscript both align with recommendations^42^ for using the SPIRIT (Standard Protocol Items: Recommendations for Interventional Trials)^43^ and CONSORT (Consolidated Standards of Reporting Trials)^44 45^ statement extension for reporting pilot trials. The findings will be relevant to a wide range of audiences interested in tobacco control, including researchers working in behavioural sciences and public health, graduate and postgraduate students, health professionals, advocacy organisations, policymakers and health economists. The primary means of disseminating study findings to these audiences will be publication of findings in peer-reviewed, open access scientific journals. The results will also be disseminated at local, national and international meetings and conferences on tobacco control. We will also disseminate a summary of study findings to study participants. Findings will inform new approaches that are timely and important, providing valuable evidence to help reduce children’s exposure to SHS in the home in Scotland and elsewhere.

## Supplementary material

10.1136/bmjopen-2025-107161 online supplemental file 1
