# Supplementary material for: Use of nicotine replacement therapy to create a smoke-free home: study protocol for a pilot randomised controlled trial of a smoke-free home intervention in Scotland
Source: BMJ Open. 2025 Aug 21;15(8):e107161. doi: 10.1136/bmjopen-2025-107161 (PMC12374659; doi:10.1136/bmjopen-2025-107161)
Supplement: online supplemental file 1 [file bmjopen-15-8-s001.docx]

**Supplementary file 1: Study consent form**

**Using nicotine replacement therapy (NRT) to create a smoke-free home**

**Parent telephone consent form**

NHS Ethical Approval Number: 23/WS/0153 Participant number:

| **1. Please listen to each of the following statements carefully. If you agree with each statement, please confirm this by saying ‘yes’. If you are unsure about any of the statements I read we can discuss them further.** | | **Researcher to circle response:** |
| --- | --- | --- |
| a. | I confirm that I understand the information sheet [v1.3/11.12.24] explaining the research study, from reading it or by talking to a researcher.  I have had the chance to think about whether I want to take part and ask questions about the study. | YES / NO  YES / NO |
| b. | I understand that my participation is my choice. I know that I can stop taking part at any time during the study. | YES / NO |
| c. | I understand that I will be allocated to one of two groups if I take part. If I am in Group A, I will receive my free nicotine replacement therapy by post during the 12 week study, and fortnightly telephone calls to support me to create a smoke-free home. If I am in Group B, I will receive current advice on creating a smoke-free home, and free nicotine replacement therapy by post at the end of the 12 week study. | YES / NO |
| d. | I agree to discuss nicotine replacement therapy product choices with a member of staff over the phone before the study begins (Group A) or at the end of the study (Group B). | YES / NO |
| e. | I agree to air quality levels being measured in my home before and after the 12 week study, using an air quality monitor. I will return the monitor by post at no cost to me. | YES / NO |
| f. | I agree to share and discuss the child information sheet about collecting a saliva sample with my child.  I agree to a member of the study team coming to my home to show me how to carry out a saliva test with my child. I understand that they will also check my child is happy to assist with this on the day, during the visit.  I provide parental consent to collect saliva samples from my youngest child aged 5 or over to obtain measures of their exposure to second-hand smoke in the home, before and after the 12 week study. I will return these samples by post at no cost to me. | YES / NO  YES / NO  YES / NO |
| g. | If I am allocated to Group A, I agree to take part in short, fortnightly telephone calls with a member of the research team to discuss to ensure I am using nicotine replacement effectively to create a smoke-free home. | YES / NO |
| h. | I agree to be contacted by the researcher during week 6 and week 12 of the study to discuss experiences of taking part, any changes to home smoking behaviours, and ways in which this research could be improved for future studies. | YES / NO |
| i. | I understand that any information I give may be used by the research team in future publications, reports or presentations. | YES / NO |
| j. | I understand that any personal data that could be used to identify me will not be used in any publications, reports or presentations. | YES / NO |
| k. | I agree to take part in this study. | YES / NO |

| **2. (If consent to participate is obtained) I would like to receive a paper copy of this completed consent form via email or post.** | **YES / NO** |
| --- | --- |
| **3**. Date of Consent |  |

| *NOT for participant use.*  *To be completed by the researcher gaining consent:* | | Initials | Please circle one |
| --- | --- | --- | --- |
| I have explained the study to the above participant and they have indicated their willingness to take part. | |  | YES / NO |
| The participant has agreed to the following methods of SHS measurement | |  | AQM/Saliva/  Both |
| Researcher Name: |  | | |
| Researcher Signature: |  | | |
